# The glial fibrillary acidic protein promoter directs sodium/iodide symporter gene expression for radioiodine therapy of malignant glioma

**DOI:** 10.3892/ol.2012.1055

**Published:** 2012-12-03

**Authors:** WEI LI, JIAN TAN, PENG WANG, NING LI, FUHAI ZHANG

**Affiliations:** Department of Nuclear Medicine, Tianjin Medical University General Hospital, Tianjin, P.R. China

**Keywords:** malignant glioma, sodium iodide symporter, glial fibrillary acidic protein promoter, radioiodine therapy

## Abstract

Radioiodine is a routine therapy for differentiated thyroid cancers. Non-thyroid cancers may be treated with radio-iodine following transfection with the human sodium/iodide symporter (hNIS) gene. The glial fibrillary acidic protein (GFAP) promoter is an effective tumor-specific promoter for gene expression and thus may be useful in targeted gene therapy of malignant glioma. The present study used GFAP promoter-modulated expression of the hNIS gene in an experimental model of radioiodine-based treatment for malignant glioma. Cells were transfected using a recombination adeno-virus and evaluated in cells by studying the transfected transgene expression through western blot analysis, ^125^I uptake and efflux, clonogenicity following ^131^I treatment and radioiodine therapy using a U87 xenograft nude mouse model. Following transfection with the hNIS gene, the cells showed 95–70-fold higher ^125^I uptake compared with the control cells transfected with Ad-cytomegalovirus (CMV)-enhanced green fluorescent protein (EGFP). The western blotting revealed bands of ∼70, 49 and 43 kDa, consistent with the hNIS, GFAP and β-actin proteins. The clonogenic assay indicated that, following exposure to 500 *μ*Ci of ^131^I-iodide for 12 h, >90% of cells transfected with the hNIS gene were killed. Ad-GFAP-hNIS-transfected and 2 mCi ^131^I-injected U87 xenograft nude mice survived the longest of the three groups. The hNIS-expressing tumor tissue accumulated ^99m^TcO_4_ rapidly within 30 min of it being intraperitoneally injected. The experiments demonstrated that effective ^131^I therapy was achieved in the malignant glioma cell lines following the induction of tumor-specific iodide uptake activity by GFAP promoter-directed hNIS gene expression *in vitro* and *in vivo*. ^131^I therapy retarded Ad-GFAP-hNIS transfected-tumor growth following injection with ^131^I in U87 xenograft-bearing nude mice.

## Introduction

Malignant glioma is the most commonly occurring primary malignant brain tumor with a poor prognosis (1). Although conventional methods, including surgery, radiotherapy, brachytherapy and chemotherapy, are used to treat glioma, numerous deficiencies remain.

Non-thyroid cancers are able to take in radioiodine following transfection with the human sodium/iodide symporter (hNIS) gene. Tumor promoters, such as the glial fibrillary acidic protein (GFAP) and human telomerase reverse transcriptase (hTERT) promoters, limit the expression of the hNIS gene in specific cells, achieving the selective expression of targeted radioiodine therapy. In our previous study, we used hTERT promoter-modulated expression of the hNIS gene in an experimental model of radioiodine-based malignant glioma treatment. This study demonstrated the significant ^131^I-iodide-induced killing of U251 and U87 human glioma cells transfected with the hNIS gene and that the hNIS gene was only expressed in telomerase-positive U251 and U87 tumor cell lines (2). However, telomerase is highly active in >85% of human cancers, not only in glioma (3). Therefore, a glioma-specific promoter is required to accomplish the radioiodine-based treatment of malignant glioma.

GFAP is an intermediate-filament protein expressed abundantly and almost exclusively in the astrocytes of the CNS (4). Due to its specificity and abundance, GFAP has become the most commonly used marker for astrocytes and a target of anti-glioma therapy (5). The promoter region of the GFAP gene has already been cloned. The GFAP promoter (the fragment −2,163 to +47 bp relative to the transcriptional start site), consisting of 2.2 kb of 5′-flanking DNA of the human GFAP (hGFAP) gene, has been revealed to drive astrocyte-specific expression in cultured cells (6). Studies have successfully demonstrated gene therapy using a GFAP promoter-driven expression vector of the therapeutic gene, including transforming growth factor (TGF)-β1 (7), brain-derived neurotrophic factor (BDNF) (8), BAX (9) and platelet-derived growth factor β polypeptide (PDGFB) (10).

The hNIS gene belongs to the sodium/solute symporter family and mediates the Na^+^/K^+^ ATPase dependent active transport of iodide across the membranes of thyroid follicular cells (11). Using targeted transfection with the hNIS gene, undifferentiated thyroid cancer, as well as non-thyroid cancers, become able to take up iodide, potentially allowing treatment with radioiodine. The hNIS expression in non-thyroidal cells results in rapid internalization but no organification of the radioiodide. Radioiodine therefore rapidly exits hNIS-transfected non-thyroidal cells. This is the major limitation of hNIS-mediated radioiodine therapy for cancer.

In the present study, we developed and evaluated the potential functional and therapeutic effectiveness of an adenovector incorporating the hNIS gene and the GFAP promoter in glioma cell lines. The GFAP promoter restricts the expression of the transfected hNIS gene to glioma cells and thus maximizes the glioma-specific uptake, while minimizing the non-specific uptake of radioiodine.

## Materials and methods

### Cell lines

U251 and U87 human glioma cells were cultured in Dulbecco’s modified Eagle medium (DMEM; Gibco BRL, Darmstadt, Germany) with 10% fetal bovine serum (FBS) and 100 U/ml penicillin/streptomycin. MRC-5 human lung fibroblasts cells were cultured in MEM Eagle’s with Earle’s Balanced Salts (MEM-EBSS; Gibco BRL) with 10% FBS. All cells were grown at 37°C under 5% CO_2_ in air.

### Western blot analysis

An EPS 2A200 electrophoresis system (Amersham Biosciences, Piscataway, NJ, USA) was used for western blot analysis. The protein extracts were centrifuged at 4°C for 10 min and electrophoresed in bis-Tris HCl-buffered 10% sodium dodecyl sulfate polyacrylamide gels (Invitrogen, Carlsbad, CA, USA). After gel electrophoresis at 140 V for 1 h, the proteins were transferred to polyvinylidene fluoride (PVDF) membranes using electroblotting. Membranes were blocked with 5% non-fat milk overnight at 4°C and then incubated separately with goat polyclonal NIS (sc-48055; Santa Cruz Biotechnology, Inc., Santa Cruz, CA, USA), mouse monoclonal β-actin (TA-09; Novus Biologicals, Littleton, CO, USA) and goat polyclonal GFAP antibodies (sc-6171; Santa Cruz Biotechnology, Inc.) for 2 h at room temperature. The membranes were then incubated with secondary antibodies at room temperature for a further 2 h and covered with Pierce ECL Western Blotting Substrate (Thermo Fisher Scientific, Waltham, MA, USA) at room temperature for 1 min and exposed to Fuji X-ray film in a darkroom. Prestained protein molecular weight standards (Spectra Multicolor Broad Range Protein Ladder, SM1841; Fermentas, Sankt Leon-Rot, Germany) were run in the same gels to compare molecular weights and estimate transfer efficiency (12).

### Construction of recombinant plasmids

The GFAP promoter region was amplified from human genomic DNA by PCR using Platinum^®^ *Taq* DNA Polymerase High Fidelity (Invitrogen). The GFAP promoter introduced both *Mlu*I and *Nhe*I restriction sites at the 5′ and 3′ ends and occupied the region from −2,163 to +47 bp relative to the transcriptional start site (GenBank M67446), which contained the core promoter and two E-boxes (6).

The GFAP promoter PCR product was ligated with PGL3-basic vector (Promega, Madison, WI, USA; creating PGL3-GFAP) and ligated with PGL3-hNIS vector (previously constructed). This was digested by *Mlu*I and *Nhe*I (creating PGL3-GFAP-hNIS).

### Luciferase assay

The expression of the luciferase gene by the GFAP promoter in tumor cells was determined using the Dual-Glo Luciferase Assay System (Promega) according to the manufacturer’s instructions. Briefly, cells seeded in 24-well plates were exposed by transfection with recombinant luciferase reporter plasmids PGL3-GFAP and background control plasmid vector pRL-TK (Promega) for 6 h at 37°C. The cells were harvested 24 h after the transfection. Then Luciferase assays were performed using a Safire2 microplate reader (TECAN, Seestrasse, Switzerland). The PGl3-control vector, containing the SV40 promoter was used as a positive control and PGL3-basic without the promoter was used as a negative control. All experiments were performed in triplicate.

### Construction of recombinant adenovirus

The hNIS gene was PCR cloned from the PGL3-GFAP-hNIS vector (previously constructed) with *Hin*dIII and *Sal*I restriction sites at the 5′ and 3′ ends and inserted into the pDC311 plasmid (Microbix Biosystems, Mississauga, ON, Canada) and named pDC311-N. The fragment which carried the GFAP promoter was then PCR cloned from the PGL3-GFAP-hNIS, with *Eco*RI and *Hin*dIII restriction sites at the 5′ and 3′ ends, and inserted into the pDC311-N vector, creating pDC311-GN. Ad-GFAP-hNIS was produced according to the instructions provided by the manufacturer of the AdMax™ Adenoviral Vector System (Microbix Biosystems). Ad-cytomegalovirus (CMV)-hNIS (previously constructed) was amplified and purified. Ad-CMV-enhanced green fluorescent protein (EGFP) was a control vector unrelated to iodine uptake and metabolism. The recombinant virus was stored at −80°C until use.

### Transfection of U251 and U87 cell lines by adenoviral infection in vitro

The cells were seeded in 6-well plates to obtain 1×10^6^ cells per well at the time of infection and infected with the adenovirus in 1,000 *μ*l of serum-free medium for 6 h, followed by addition of 10% FBS new medium. The transfected cells were incubated for 24 h. Ad-CMV-hNIS, Ad-GFAP-hNIS and Ad-CMV-EGFP were used to infect U87, U251 and MRC-5 cells, respectively.

### Iodide uptake and efflux assays

Cells were seeded in 6-well plates and infected with the recombinant adenovirus for 6 h, then placed in fresh DMEM with 10% FBS and incubated for an additional 24 h. The radioactivity was measured with a γ counter (LKB Gamma 1261; LKB Instruments, Mt Waverly, Australia). To measure the ^125^I uptake, 1×10^6^ cells per well were cultured with 1 ml 10% FBS-DMEM (containing 0.5 *μ*Ci Na^125^I) for 0, 10, 20, 30 and 40 min. The ^125^I-containing medium was then decanted, cells were washed twice with PBS, lysed with 0.3 mol/l sodium hydroxide and counted. To measure the ^125^I efflux, 1×10^6^ cells per well were cultured for 1 h with 1 ml 10% FBS-DMEM (containing 0.5 *μ*Ci Na^125^I), then the ^125^I-containing medium was decanted. After the cells were washed twice with PBS, fresh non-radioactive medium was added to the 6-well plates. The cells were then cultured again for 0, 5, 10, 15 and 20 min. Subsequently, the cells were washed, lysed and counted. All experiments were performed in triplicate (11).

### Cell killing with ^131^I and clonogenic assay in vitro

Cells were seeded in 6-well plates and infected with the adenovirus for 6 h and then placed in fresh DMEM with 10% FBS for an additional 24 h, washed twice with PBS and incubated with 1 ml of DMEM with 10% FBS, containing 500 *μ*Ci ^131^I. After a 12-h incubation, cells were washed twice with PBS. For each condition [Ad-GFAP-hNIS, Ad-CMV-hNIS and Ad-CMV-EGFR (the control adenovirus) infected], cells were plated in 24-well plates at densities of 100 cells/well and incubated for 1 week at 37°C. The cells were then washed twice with PBS, fixed with 0.5 ml Carnoy’s solution (a freshly prepared 3:1 mixture of methanol and acetic acid) and stained with a crystal violet solution (for 250 ml, 0.5 g crystal violet, 25 ml 40% formaldehyde, 50 ml ethanol and 175 ml water). Colonies of >20 cells were counted. All experiments were performed in triplicate (13,14).

### Animal model

Experiments involving animals were performed with the approval of the Beijing Experimental Animal Center of Peking Union Medical, China. The generation of subcutaneous tumors was performed as follows (11): 5×10^6^ U87 tumor cells were transplanted subcutaneously into the right shoulder of 4-week-old BALB/c female nude mice weighing 190*–*210 g. When the tumors had reached a minimum size of 10 mm in diameter (∼3 weeks after cell injection), the recombinant adenovirus was injected into the tumor (5×10^9^ PFU in 50 *μ*l of PBS). U251 and U87 are to glioma cell lines and the data for the U87 and U251 cells were similar to the *in vitro* clonogenic assay, so U87 cells were selected as representative for animal testing.

### Radioiodine therapy study in vivo

The 5×10^9^ PFU in 50 *μ*l of PBS recombinant adenovirus was injected into the tumor when the tumor grew to a minimum of 10 mm in diameter and 1 day later, 2 mCi ^131^I was intraperitoneally injected. The tumor size was monitored prior to the administration of radio-iodine and every 7 days thereafter by measuring 3 diameters with a sliding caliper and converting them to volume using the formula V=4πabc/3. For the therapeutic experiments, all rats were divided into 2 groups in terms of Ad-GFAP-hNIS and Ad-CMV-EGFP (the control adenovirus). Each group was then divided into 2 subgroups in terms of ^131^I administration (3).

### Tumor imaging

For imaging studies, only animals bearing tumors with a minimum size of 10 mm in diameter were accepted. The Ad-GFAP-hNIS (5×10^9^ PFU in 50 *μ*l of PBS) was injected into the tumor, then 1 mCi of ^99m^TcO_4_ was intraperitoneally injected 1 day later and 20 min after that, images were obtained using the Gamma Camera (Discovery VH; GE Healthcare, Piscataway, NJ, USA) (15).

### Statistical analysis

All experiments were performed in triplicate unless otherwise indicated. Statistical analysis was performed using SPSS software (SPSS 13.0; Tianjin, China). The results are presented as the mean ± SD. Statistical significance was tested using the student t-test procedure. P<0.05 was considered to indicate a statistically significant result.

## Results

### Western blot analysis

**Figure 1.**
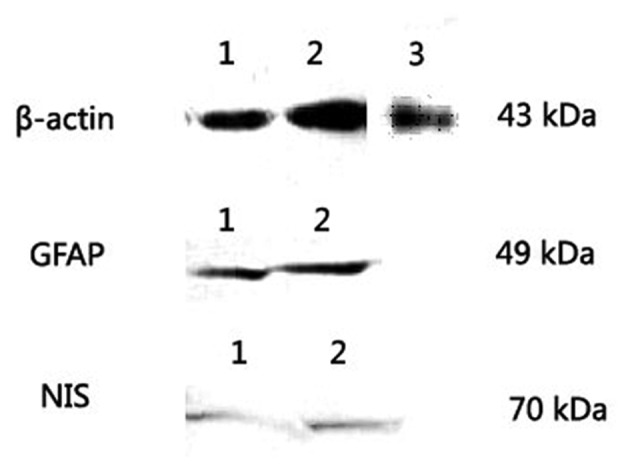
Western blot analysis. 1, U251 cell line; 2, U87 cell line; 3, MRC-5 cell line; GFAP, glial fibrillary acidic protein; NIS, sodium/iodide symporter.

The GFAP and hNIS protein levels were analyzed by western blotting. In U87, U251 and MRC-5 cells, the western blotting showed that the GFAP protein was expressed in the GFAP-positive U251 and U87 tumor cell lines as a major band of 49 kDa but not in the GFAP-negative MRC-5 cells, since MRC-5 was a normal cell line. The β-actin protein was used as a positive control and expressed in the U251, U87 and MRC-5 cells as a major band corresponding to a molecular weight of 43 kDa (Fig. 1). Following transfection with Ad-GFAP-hNIS, the protein level of the hNIS gene was analyzed by western blotting in the U251 and U87 tumor cells and MRC-5 normal cells. The hNIS protein was detected as a major band corresponding to a molecular weight of 70 kDa in U251 and U87 tumor cell lines but was not expressed in the GFAP-negative MRC-5 cell line (Fig. 1).

### Luciferase assay

**Figure 2.**
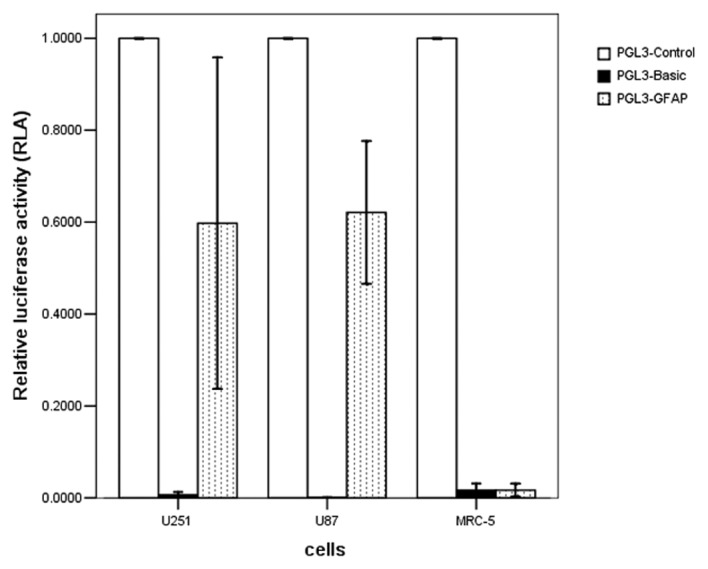
Transcriptional activity in U251, U87 and MRC-5 cell lines. The RLA of plasmids PGL3-GFAP, PGL3-basic and PGL3-control was compared. The luciferase activity ratio in each plasmid was first compared with the background control pRL-TK vector. The RLA for each experimental treatment was calculated using the following formula: RLA = (experimental sample ratio - negative control ratio) / (positive control ratio - negative control ratio). The negative control was PGL3-basic and the positive control was PGL3-control. The RLA of the PGL3-control was ∼1, while the RLA of PGL3-basic was ∼0. The data are the mean ± SD from three independent experiments. GFAP, glial fibrillary acidic protein; RLA, relative luciferase activity.

To assess the cell-specific transcriptional activity of the GFAP promoter, a reporter gene assay using a luciferase assay was performed in transiently transfected cells. The transient transfection showed that the GFAP promoter is able to cause luciferase gene expression in GFAP-positive U251 and U87 tumor cell lines, without expression in the GFAP-negative MRC-5 cells. The transcriptional activity of the GFAP promoter (PGL3-GFAP) reached 59.75±0.34 and 62.1±0.6% of that in the positive-control cells with the SV40 promoter (PGL3-control) in the U251 and U87 cells (Fig. 2).

### Iodide uptake and efflux assays

**Figure 3.**
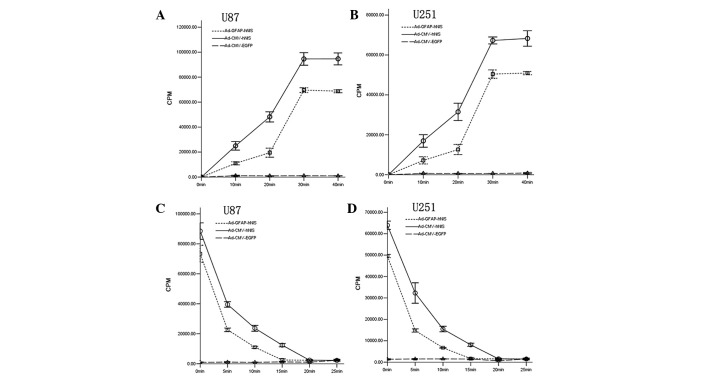
Iodide uptake and efflux assays in U87 and U251 cell lines. (A) Iodide uptake assays in U87 cell lines. Maximal ^125^I iodide uptake of U87 transfected with Ad-CMV-hNIS and Ad-GFAP-hNIS was 95.1- and 69.5-fold higher, respectively, than the control cells (transfected with Ad-CMV-EGFP). All four experimental cell lines reached the maximum within 30 min when radioiodine was present in the medium. (B) Iodide uptake assays in U251 cell lines. Maximal ^125^I iodide uptake of U251 transfected with Ad-CMV-hNIS, and Ad-GFAP-hNIS was 94.7- and 79.8-fold higher, respectively, than the control cells. (C) Iodide efflux assays in U87 cells. The efflux of radioiodide from U87 cells was rapid and the biological half-times were short. The biological times of iodide in U87 cells transfected with Ad-CMV-hNIS, with Ad-GFAP-hNIS were 17.8 and 10.9 min, respectively. The biological half-life of the iodide efflux assays in this study was based on the cubic or inverse model on SPSS 13.0. (D) Iodide efflux assays in U251 cells. The efflux of radioiodide from the U251 cells was also rapid. The biological times of iodide in U251 cells transfected with Ad-CMV-hNIS and Ad-GFAP-hNIS were 16.3 and 10.3 min, respectively. CMV, cytomegalovirus; hNIS, human sodium/iodide symporter; GFAP, glial fibrillary acidic protein; EGFP, enhanced green fluorescent protein.

To determine the iodide uptake and efflux, ^125^I-timed activity measurements were performed in U251 and U87 human glioma cells. Radioiodide uptake in the adenovirus-transfected U251 and U87 cells was rapid (with the exception of Ad-CMV-EGFP), reaching maximal levels within 30 min and the Ad-CMV-hNIS-transfected cells had the highest rate (Fig. 3A and B). To determine the iodide efflux, the iodide uptake was permitted to proceed for 1 h, so the steady-state level of accumulation was achieved. Following replacement of the ^125^I-containing medium with nonradioactive medium, the intracellular iodide was continuously released into the medium and a rapid efflux of radioiodine from cells was evident (Fig. 3C and D). ^125^I has a long physical half-life of 60 days, so the effect of decay over the 20- to 50-min duration of the experiments was ignored.

### Clonogenic assay in vitro

**Figure 4.**
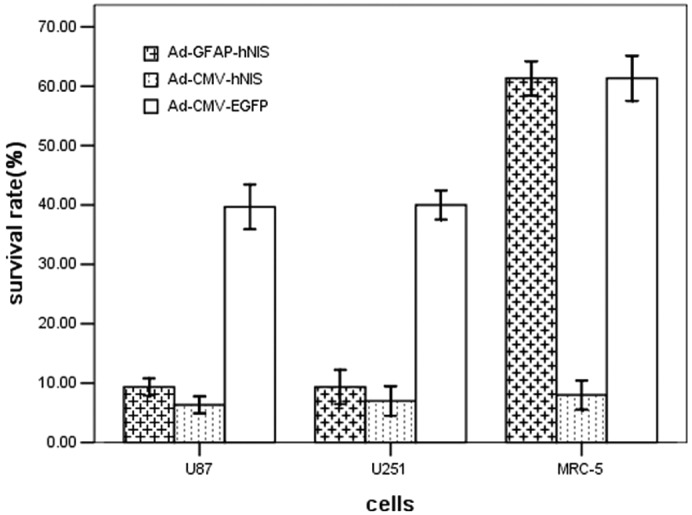
Clonogenic assay of U87 and U251 tumor cells and MRC-5 normal cells. Following 500 *μ*Ci ^131^I treatment in the U87 cells, the clone formation rates were 6.33±0.58% in the Ad-GFAP-hNIS-transfected group and 9.31±0.5% in the Ad-CMV-hNIS-transfected group. In the corresponding U251 cell groups, the rates were 7.0±1.0 and 9.33±1.15%, respectively. GFAP, glial fibrillary acidic protein; hNIS, human sodium/iodide symporter; CMV, cytomegalovirus; EGFP, enhanced green fluorescent protein.

The clonogenic assay investigated whether ^131^I showed selective cytotoxic activity in the hNIS gene-expressing cells. The survival rates of transfected U251, U87 and MRC-5 cells not incubated with ^131^I were ∼90%. The survival rates of the CMV promoter-transfected U251 and U87 tumor cells and MRC-5 cells were all lower compared with the GFAP promoter-transfected cells. Since the GFAP promoter was unable to express the hNIS gene in normal MRC-5 cells, the survival rates of the cells transfected with the GFAP promoter adenovirus and transfected with Ad-CMV-EGFR (control empty adenovirus) were similar. Transfection with the hNIS, whether with the GFAP or CMV promoter, resulted in more ^131^I-induced cell killing than without hNIS gene transfection (Fig. 4).

### Radioiodine therapy study in vivo

**Figure 5.**
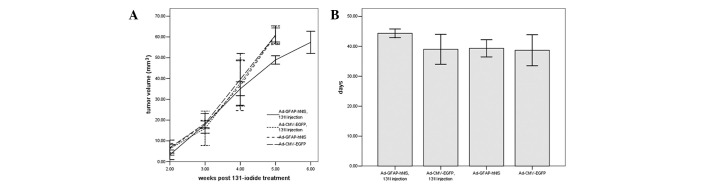
^131^I therapy in nude mice bearing U87 tumors. (A) The changes in tumor volumes in U87 xenograft nude mice. (B) The life spans of U87 xenograft nude mice.

After the U87 xenografts were injected with adenovirus, the group injected with Ad-GFAP-hNIS (5×10^9^ PFU in 50 *μ*l of PBS) and 500 *μ*Ci ^131^I survived the longest of the three groups, demonstrating that the ^131^I therapy was capable of increasing the U87 xenograft nude mice survival. The three groups (Ad-CMV-EGFP and ^131^I, Ad-CMV-EGFP and Ad-GFAP-hNIS injection) survived for similar periods, showing that the adenovirus injection and 500 *μ*Ci ^131^I injection had no effect on the nude mice’s life span (Fig. 5). Moreover, after the U87 xenografts were injected with 2 mCi ^131^I, the ^131^I therapy retarded the Ad-GFAP-hNIS transfected-tumor growth, whereas the U87 tumors grew significantly after transfection with Ad-CMV-EGFP since they were unable to take up ^131^I; Fig. 5). Since the data for the U87 and U251 cells were similar in the *in vitro* clonogenic assay, only the U87 cells were selected to use in the the radio-iodine therapy study *in vivo*.

### Tumor imaging

**Figure 6.**
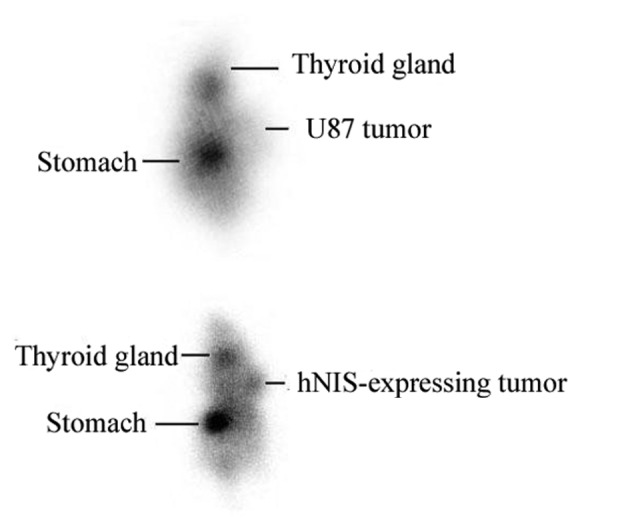
^99m^TcO_4_ scans of nude mice. hNIS, human sodium/iodide symporter.

The hNIS-expressing tumor tissue accumulated ^99m^TcO_4_ rapidly when ^99m^TcO_4_ was intraperitoneally injected 20 min later, whereas the control tumor injected with the control virus (Ad-CMV-EGFP) was not visualized (Fig. 6). Normal NIS-expressing tissues, including those of the thyroid gland and stomach, were also clearly visible.

## Discussion

Radioiodine therapy for differentiated thyroid carcinoma has been used for a number of years. Through targeted transfection and expression of the hNIS gene, non-thyroid cancers become able to take in iodine and so respond to radioiodine therapy in the same manner as thyroid cancer. Moreover, due to the crossfire effect of radiation therapy, radioiodine may kill not only the NIS-expressing cells but also adjacent tumor cells. However, if the hNIS gene was expressed in all transfected cells, the therapeutic gene would affect tumor and normal cells. Using a tumor-specific promoter system is likely to solve this problem and the tumor-specific transfection of the hNIS gene has been applied to a variety of tumors, including thyroid, prostate, colon, breast, liver and lung cancer (11,13,15,16). Studies have demonstrated that the α-fetoprotein (AFP) (15), carcinoembryonic antigen (CEA) (17), ubiquitin C (UbC) (18), murine albumin (mAlb) (11), prostate-specific antigen (PSA) (12) and the glucose transporter gene 1 (GTI-1.3) promoters (19) all lead to tumor-specific hNIS expression. However, the tumor-specific promoters are often useful only for the particular types of cancer from which they were derived and generally less effective than non-specific or constitutive promoters such as the CMV and simian virus 40 (SV40) promoters.

In the present study, we constructed and evaluated the potential functional and therapeutic effectiveness of a recombinant adenovirus Ad-GFAP-hNIS carrying the hNIS gene controlled by the GFAP promoter in U251 and U87 glioma cell lines. The cells transfected with the hNIS gene under the control of the tumor-specific GFAP promoter, took in radioiodine and had lower survival rates for ^131^I-treated U251 and U87 cells compared with the control cells (transfected with Ad-CMV-EGFP) *in vitro. In vivo*, the radioiodine therapy study showed that the U87 xenografts injected with Ad-GFAP-hNIS and 2 mCi ^131^I survived the longest of the three groups (Ad-CMV-EGFP and ^131^I, Ad-CMV-EGFP and Ad-GFAP-hNIS injection). ^131^I injection had similar data to the other three groups (not shown in Fig. 6), so the adenovirus and ^131^I injection had no effect on the life spans of the nude mice and was able to accumulate ^99m^TcO_4_ successfully in the ^99m^TcO_4_ scans. In the studies of tumor-specific promoters, the GFAP promoter exhibited glioma-specific hNIS expression in the U87 and U251 cells and did not cause hNIS gene expression in MRC-5 normal cells.

The data showed that glioma-specific radioiodine intake was caused by transfection with the hNIS gene under the control of the GFAP promoter, but the GFAP promoter may also have been activated by normal astrocytes in the CNS. Identifying how to increase the selectivity of the GFAP promoter using dual promoters to restrict the gene expression may be a way to solve this problem. Furthermore, Doloff *et al* constructed an adenovirus that used DF3/Muc1 and hTERT tumor-specific promoters to drive separate E1A expression and exhibited improved oncolysis in numerous cancer cell lines (20). Löw *et al* developed a dual promoter lentiviral vector in which the EGFP gene was expressed from the CMV-enhanced chicken β-actin (CAG) promoter and copGFP was expressed from the elongation factor-1α (EF-1α) promoter (21). These dual-promoter studies drove the expression of same or different genes, respectively.

In conclusion, hNIS gene expression mediated by the GFAP promoter was restricted to only GFAP-positive cells. Nude mice harboring U87 xenografts transfected with Ad-GFAP-hNIS and injected with ^131^I survived the longest of the three groups and were able to accumulate ^99m^TcO_4_ successfully in the ^99m^TcO_4_ scans.
